# Editorial: Self-concept plasticity: behavioral and neural evidence

**DOI:** 10.3389/fnhum.2025.1584910

**Published:** 2025-03-24

**Authors:** Mateusz Woźniak, Mariët van Buuren, Pawel Tacikowski

**Affiliations:** ^1^Social Cognition in Human Robot Interaction Group, Italian Institute of Technology, Genoa, Italy; ^2^Social Mind and Body Group, Department of Cognitive Science, Central European University, Vienna, Austria; ^3^Department of Clinical, Neuro and Developmental Psychology, Faculty of Behavioral and Movement Sciences, Vrije Universiteit Amsterdam, Amsterdam, Netherlands; ^4^Coimbra Institute for Biomedical Imaging and Translational Research, Coimbra University, Coimbra, Portugal; ^5^Department of Neuroscience, Karolinska Institutet, Stockholm, Sweden

**Keywords:** self-concept, bodily self, self-plasticity, self-development, full-body illusion, better-than-average effect

Self-concept refers to an individual's idea of who they are as a person. This complex mental representation falls under the even broader notion of the self, which also encompasses the bodily aspects of being an embodied agent with a first-person perspective. The way we think and feel about ourselves is not fixed over time. This is evident when examining the self across development (Bertenthal and Fischer, 1978; de Klerk et al., 2021; Harter, 2012; Riva, 2018)—we are not the same people we were as children, and even in adulthood, our self can change dramatically following life events such as becoming a parent or undergoing psychotherapy. Certain aspects of the self can also change on much shorter timescales—within minutes or even seconds—as demonstrated by research on bodily illusions (Blanke and Metzinger, 2009; Botvinick and Cohen, 1998; Ehrsson, 2020; Petkova and Ehrsson, 2008; Tsakiris and Haggard, 2005) and the self-prioritization effect (Sui et al., 2012; Sui and Humphreys, 2015). Thus, the plasticity of the self is not a singular phenomenon but rather a complex, multifaceted process. This diversity is the primary motivation behind this Research Topic.

In a theoretical article, Woźniak approached the issue of self-plasticity from both Bayesian and developmental perspectives. First, he proposed that the self can be understood as a collection of self-models that enable us to classify our perceptions and other mental content as either representing the self or not. These self-models underpin our capacity to distinguish ourselves from others and objects in the world via Bayesian inference. Second, he proposed a developmental trajectory, as well as the mechanism inspired by Susan Carey's work (Carey, 2009), through which people acquire new self-models over the course of their lives. In his paper, he also outlined a proposal of how self-representations become progressively more abstract, starting from the primordial self-other distinction, through the formation of several aspects of the bodily self, up until the formation and further refinement of the social, extended, and abstract selves.

In relation to the embodied aspects of the self, Yamamoto and Nakao investigated how cognitive factors influence the fundamental feeling of body ownership—the sense that our body belongs to us. Using head-mounted displays, participants viewed an avatar's back being touched with an object while experiencing either synchronous or asynchronous touches on their own back delivered by the experimenter. Consistent with previous findings, the study confirmed that visuotactile synchrony, but not asynchrony, was linked to the illusory perception that the avatar's body, seen from a third-person perspective, was their own. However, the key finding was that the strength of this full-body illusion decreased when participants were instructed to imagine the virtual body as their own while experiencing abdominal pain. This study contributes to our understanding of how higher-order cognitive processes—specifically, negative interpretations—can modulate perceptual bodily experiences.

Żochowska et al. explored the plasticity of the self by comparing attentional processing of one's own face with arbitrary information linked to the self. To investigate the latter, they asked participants to associate different geometrical figures with themselves, their best friend, or a stranger. Using electroencephalography, they examined whether perceiving self-associated shapes affects spatial attention in the same way as viewing one's own face. They found that in the dot-probe task, both participants' faces and self-associated shapes modulated the N2pc ERP component. A similar effect was observed for participants' friends' faces but not for shapes associated with those friends.

Moving on to social aspects of the self, Ding and Sugiura investigated the better-than-average effect (BTAE)—the phenomenon in which individuals perceive themselves as better than the average person. This online survey study, conducted on young Japanese adults, assessed subjective evaluations of the self and the “average other” based on trait adjectives related to either positive or negative moral attributes (e.g., warmhearted or heartless), or to positive or negative competence attributes (e.g., smart or uneducated). The results indicated that the BTAE occurred only under the negative moral condition (“I am not as heartless as the average person”), whereas the worse-than-others effect was observed in both competence conditions (e.g., “I am not as smart, and I am more uneducated than the average person”). These findings suggest that the BTAE may not be as universal as previously thought and that sociocultural dynamics may influence this effect differently across various domains.

In the final paper of the Research Topic, Schmautz et al. approached plasticity of the self from yet another angle. They tackled the experiences of losing the self, or experiencing one's self as unified with others and the world. They developed the OCEANic scale to measure such feelings and conducted factor analyses to validate it. These analyses uncovered two factors. The first factor represented oceanic experiences that were perceived in negative terms, such as feeling of drowning, fragmentation of the self, or all-enveloping darkness. The second, positive, factor represented experiences of having merged or united with others, which were understood by participants as something good and enriching. Moreover, the authors investigated how these two factors related to personality traits, as well as affective neurobiological traits such as fear, sadness, lust, play, etc. They suggest that their questionnaire can be used in future psychological and neuroscientific studies investigating such atypical changes to the self.

The Research Topic demonstrates that the plasticity of the self can be explored from a wide range of perspectives, including developmental theory, experimental research using multisensory bodily illusions, electrophysiological studies on arbitrary self-associations, social psychology research on self-comparison, and questionnaire-based approaches to measuring atypical self-experiences. On the one hand, this diversity of approaches highlights numerous potential pathways for future research. On the other hand, it presents terminological and methodological challenges, emphasizing the need for synthesis and interdisciplinary dialogue. Such dialogue is essential for advancing our understanding of the fundamental mechanisms that shape the self over time, both in the short and long term.
